# Fine Mapping of *fw6.3*, a Major-Effect Quantitative Trait Locus That Controls Fruit Weight in Tomato

**DOI:** 10.3390/plants12112065

**Published:** 2023-05-23

**Authors:** Yu Ning, Kai Wei, Shanshan Li, Li Zhang, Ziyue Chen, Feifei Lu, Pei Yang, Mengxia Yang, Xiaolin Liu, Xiaoyan Liu, Xiaotian Wang, Xue Cao, Xiaoxuan Wang, Yanmei Guo, Lei Liu, Xin Li, Yongchen Du, Junming Li, Zejun Huang

**Affiliations:** State Key Laboratory of Vegetable Biobreeding, Institute of Vegetables and Flowers, Chinese Academy of Agricultural Sciences, Beijing 100081, China

**Keywords:** tomato, fruit weight, *fw6.3*, fine mapping, soluble solids content, overdominance

## Abstract

Tomato (*Solanum lycopersicum*) is a widely consumed vegetable, and the tomato fruit weight is a key yield component. Many quantitative trait loci (QTLs) controlling tomato fruit weight have been identified, and six of them have been fine-mapped and cloned. Here, four loci controlling tomato fruit weight were identified in an F_2_ population through QTL seq.; *fruit weight 6.3 (fw6.3*) was a major-effect QTL and its percentage of variation explanation (R^2^) was 0.118. This QTL was fine-mapped to a 62.6 kb interval on chromosome 6. According to the annotated tomato genome (version SL4.0, annotation ITAG4.0), this interval contained seven genes, including *Solyc06g074350* (the *SELF-PRUNING* gene), which was likely the candidate gene underlying variation in fruit weight. The *SELF-PRUNING* gene contained a single-nucleotide polymorphism that resulted in an amino acid substitution in the protein sequence. The large-fruit allele of *fw6.3* (*fw6.3_HG_*) was overdominant to the small-fruit allele *fw6.3_RG_*. The soluble solids content was also increased by *fw6.3_HG_*. These findings provide valuable information that will aid the cloning of the *FW6.3* gene and ongoing efforts to breed tomato plants with higher yield and quality via molecular marker-assisted selection.

## 1. Introduction

Tomato (*Solanum lycopersicum*) is an economically important vegetable, as well as a model plant for fleshy fruit physiology and development research. A major goal of tomato breeding programs is maximizing fruit yield, especially fruit weight, which is a quantitative trait. Many quantitative trait loci (QTLs) affecting tomato fruit weight have been identified using classical approaches, such as QTL mapping, QTL-seq, and genome-wide association studies [1,2,3].

To date, a total of six loci affecting tomato fruit weight have been fine-mapped and cloned, including *fruit weight2.2* (*fw2.2*) [4], *fw3.2* [5], *fw11.3* [6], *fasciated* (*fas*) [7,8], *locule number* (*lc*) [9,10], and *globe* [11]. *fw2.2* was the first QTL controlling fruit weight to be cloned from any vegetable or fruit crop. This QTL is located on the long arm of chromosome 2. The *FW2.2* gene encodes a protein that negatively regulates cell division. It is supposed that DNA variation(s) in the promoter sequence reduces the expression of *FW2.2*, which promotes cell division and increases fruit size [4]. Fine mapping of *fw3.2* has revealed that it occurred on chromosome 3 in a 24.4 kb interval. *FW3.2* was named *SlKLUH* because it is an ortholog of *KLUH*, which is a member of the P450 family [5,12,13]. A single-nucleotide polymorphism (SNP) in the promoter region of the *SlKLUH* gene has been shown to be highly associated with fruit weight [5]. A recent study revealed that an approximately 50 kb tandem duplication at the *fw3.2* locus increases the expression of the *SlKLUH* gene and the number of cells in fruit [5,14]. The *fw11.3* locus affects fruit weight by mediating increases in cell size. Fine mapping of *fw11.3* has revealed that it occurs on chromosome 11 in a 13 kb interval. The large-fruit allele of *FW11.3* encodes a shorter protein with 194 fewer amino acids than the small-fruit allele due to a 1.4 kb deletion, and it results in an increase in the size of fruit cells and, thus, fruit size. *FW11.3* has, therefore, been named a cell size regulator [6,15].

The loci *fas* and *lc* affect the number of locules and fruit weight [16]. *LC* encodes *SlWUS*, and two SNPs 1080 bp downstream of this gene affect the expression of *SlWUS* [9,10]. *FAS* encodes *SlCLV3*, and a 294 kb inversion down-regulates the expression of *SlCLV3*, which results in an increase in the number of locules [8]. The *globe* locus affects various dimensions of fruit, including fruit length. Fine mapping of *globe* has revealed that it occurs on chromosome 12 in a 392 kb interval. The *GLOBE* gene encodes a brassinosteroid hydroxylase. A single base insertion in the coding sequence of this gene results in a frameshift mutation [11].

The dominance effect and overdominance effect are important contributors to heterosis that have been widely used in tomato breeding [17]. Among the above-mentioned six loci, the large fruit alleles of *fw3.2* and *fw11.3* show partial dominance over their small fruit alleles [5,13,15], whereas, the large fruit alleles of *fw2.2*, *fas*, *lc*, and *globe* are partially recessive to their small fruit alleles [4,9,10,11]. The use of these loci has, thus, been limited in the heterosis breeding of large tomato fruit. Additional studies are needed to identify major-effect fruit weight loci, whose large-fruit alleles are completely dominant or overdominant to their small-fruit alleles.

In this study, four QTLs affecting tomato fruit weight were identified, including *fw6.3*, which was a major-effect QTL and showed an overdominant action. *fw6.3* also led to increases in the soluble solids content (SSC). Fine mapping of *fw6.3* revealed that it occurs on chromosome 6 in a 62.6 kb interval. The putative candidate gene of *FW6.3* was *SELF PRUNING* (*SP*). The *SP* gene in the *fw6.3* locus contained an SNP that resulted in an amino acid substitution in the protein sequence. The results of this study provide new insights that enhance our understanding of the mechanism underlying the regulation of tomato fruit weight at the molecular level. Our findings will also aid the breeding of tomato varieties with higher yields and quality.

## 2. Results

### 2.1. Variation in Fruit Weight in the Segregating Population

**Figure 1:**
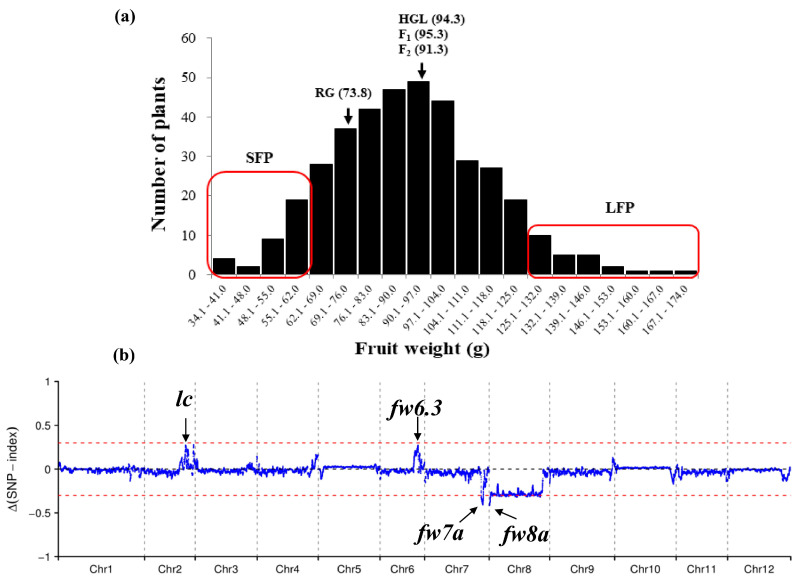
Identification of QTLs associated with tomato fruit weight. (**a**) Distribution of fruit weight in the F_2_ population derived from the cross between RG and HGL. (**b**) Average ΔSNP-index (difference between the SNP-index of the SFP and the SNP-index of the LFP) calculated via sliding window analysis. The blue line shows the ΔSNP-index; red dotted lines correspond to the |ΔSNP-index| threshold of 0.3. SFP: small fruit pool; LFP: large fruit pool; RG and HGL: two parents of the F_1_.

The average tomato fruit weight of Rio Grande (RG) HGL (an offspring of plant 11S74-3 that was used in the fine-mapping of *fw11.3* and derived from a cross between Howard German (HG) and LA1589) [6], and their F_1_ plants was 94.3 g, 73.8 g, and 95.3 g, respectively (Figure 1a). The fruit weight of the F_2_ population ranged from 34.1 g to 174.0 g (Figure 1a). These data indicate that fruit weight was quantitatively inherited in the F_2_ population.

### 2.2. QTL Analysis of Tomato Fruit Weight

QTL-seq was conducted on the large fruit pool (LFP) and small fruit pool (SFP) from the F_2_ population. The LFP comprised the 20 plants with the highest fruit weight (121.8 g to 167.9 g), and the SFP comprised the 20 plants with the lowest fruit weight (34.2 g to 58.6 g) (Figure 1a). The Δ(SNP-index) plot was generated by taking the difference between the SNP-index of the SFP and the SNP-index of the LFP (Figure 1b). Four candidate QTLs associated with fruit weight were identified. These QTLs were located on chromosomes 2, 6, 7, and 8 and were referred to as *lc*, *fw6.3*, *fw7a*, and *fw8a*, respectively (Figure 1b). The candidate QTL located on chromosome 2 was named *lc* because HGL contained the two SNPs associated with the *lc* locus, which enhances fruit weight [10] according to the resequencing data. The candidate QTL located on chromosome 6 was named *fw6.3* because its position was the same as that of the *fw6.3* locus identified in previous studies [2,18,19].

Several markers within the candidate QTL regions were used to genotype the 388 plants in the F_2_ population to confirm the association of these QTLs with variation in fruit weight revealed by QTL-seq. Our findings suggested that the *lc* and *fw6.3* loci contained alleles that conferred a high fruit weight from HGL, whereas the *fw7a* and *fw8a* loci contained alleles that conferred a high fruit weight from RG. *lc* and *fw6.3* were major-effect QTLs (R^2^ ≥ 0.1). The amount of phenotypic variation explained by these two QTLs (according to R^2^ values) was 0.115 and 0.118, respectively (Table S1).

### 2.3. Fine Mapping and Analysis of Candidate Genes of the fw6.3 Locus

**Figure 2:**
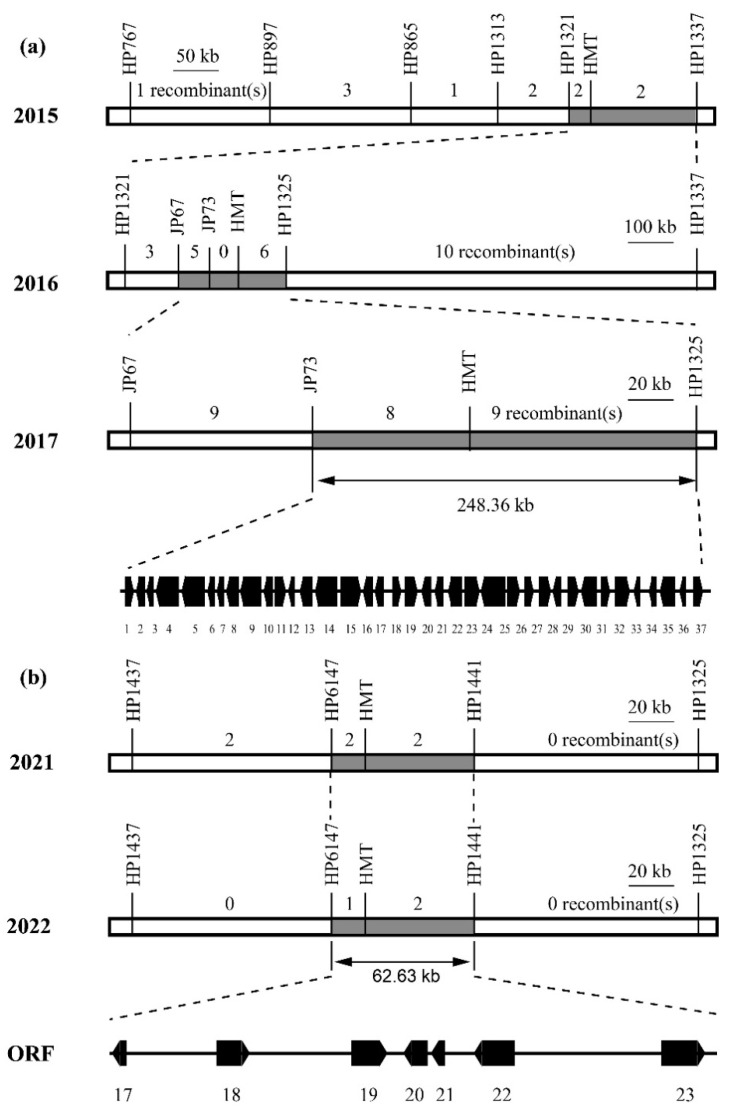
Fine mapping of the *fw6.3* locus. (**a**) Results of recombinant progeny tests of the population derived from RG and HGL. (**b**) Results of recombinant progeny tests of the population derived from RG and LA1589.

Progeny tests were conducted in the spring of 2015 on 10 recombinants from the F_2_ population with crossover sites that were located close to the HMT marker on chromosome 6 and with homozygous genotypes in intervals containing other detected fruit weight loci. Progeny tests revealed that the *fw6.3* locus was located in a 1.66 Mb region on chromosome 6 between the markers HP1321 and HP1337 (Figure 2a, Table S2). In 2016, the *fw6.3* locus was fine-mapped to a region between markers JP67 and HP1325 (Figure 2a, Table S3); in 2017, the *fw6.3* locus was fine-mapped to a 248.8 kb region between markers JP73 and HP1325 (Figure 2a, Table S4). This interval contained 37 genes (Table S5). These resequencing data showed that there is no sequence polymorphism between HGL and HG in the entire 248.8 kb region, which suggests that the allele of the *fw6.3* locus that enhances fruit size was derived from HG. The development of new molecular markers to screen recombinants is difficult due to the low amount of sequence variation, with the exception of a large, high copy number fragment in the *fw6.3* candidate region between RG and HG. However, the number of sequence polymorphisms was much higher between HG and LA1589 (a line of *S. pimpinellifolium* with very small fruit); consequently, populations obtained from the cross between near-isogenic line (NIL)-*fw6.3_HG_* and NIL-*fw6.3_LA1589_* were used for the fine mapping of *fw6.3*. According to the results of the progeny tests, no significant difference in fruit weight was observed between the allele of *fw6.3* derived from HG and that derived from LA1589 (Table S6); this suggests that both HG and LA1589 contained the large-fruit allele of *fw6.3*.

Populations obtained from the cross between NIL-*fw6.3_RG_* and NIL-*fw6.3_LA1589_* were used to further narrow the interval containing *fw6.3*. The *fw6.3* locus was fine-mapped to a 62.6 kb interval between markers HP6147 and HP1441 in the spring of 2021 and 2022 (Figure 2b, Tables S7 and S8). According to the reference genome of the tomato genome annotation database (ITAG release 4.0) in the Sol Genomics Network (SGN) (https://solgenomics.net/), seven putative genes were detected in this 62.6 kb interval (Table S9). Many sequence polymorphisms between RG and LA1589 were detected, including one SNP in the *SP* gene. This SNP has been previously reported to result in an amino acid substitution [20]. In the entire 62.6 kb interval, with the exception of the SNP in the *SP* gene, there are two InDels (a single nucleotide A insertion after SL4.0ch06: 43674162 and a single nucleotide A insertion after SL4.0ch06: 43676959) between RG and HG. However, these two InDels were not present between RG and LA1589. So, the *SP* gene was speculated to be the putative candidate gene for the *fw6.3* locus.

### 2.4. Gene Action of fw6.3

The d/a value of the *fw6.3* locus was 1.02 in the F_2_ population (Table S1), which suggests that the large-fruit allele of *fw6.3* was dominant to the small-fruit allele. The gene action of *fw6.3* was further analyzed using high-generation populations. The d/a values of the *fw6.3* locus in the five populations (two F4, one BC2S4, one BC4S6, and one BC7S4) were all larger than 1.25 (1.27 to 1.78) (Table 1 and Table S10), which indicates that the large-fruit allele *fw6.3_HG_* was overdominant to the small-fruit allele *fw6.3_RG_*.

### 2.5. Fruit Diameter and Length

**Figure 3:**
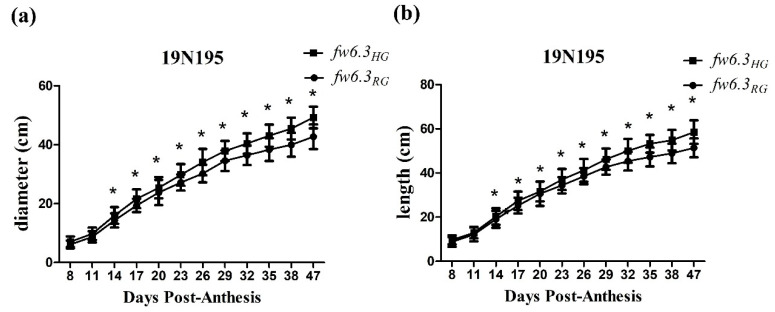
Changes in fruit size during fruit development. (**a**) Fruit diameter. (**b**) Fruit length. Data points correspond to the mean diameter and length of developing fruits from 10 plants of each line. A Student’s *t*-test was conducted, and black asterisks (* *p* < 0.05) represent significant differences.

To determine the basis of the fruit weight difference affected by the *fw6.3* locus, three fruit size and shape attributes (fruit diameter, fruit length, and fruit shape index) were examined. The fruit diameter and length were higher in mature fruit with the large-fruit allele *fw6.3_HG_* compared with the fruit that contained the small-fruit allele *fw6.3_RG_*; however, no difference in the fruit shape index was observed between fruits with *fw6.3_HG_* and with *fw6.3_RG_* (Table 2 and Table S11). To elucidate fruit size differences determined during fruit development, fruit diameter and length were measured from 8 days post-anthesis (DPA) to the red ripening stage. Both the diameter and length of fruit containing *fw6.3_HG_* were significantly larger than the diameter and length of fruit containing *fw6.3_RG_* (Figure 3) starting at 14 DPA, which suggests that *fw6.3* affected fruit size starting at the early stage of fruit development.

### 2.6. Flower and Leaf Removal Experiment

The growth habit of NIL-*fw6.3_HG_* and NIL-*fw6.3_RG_* differed. NIL-*fw6.3_HG_* was characterized by indeterminate growth, and NIL-*fw6.3_RG_* was characterized by determinate growth. The number of leaves was higher in NIL-*fw6.3_HG_* than in NIL-*fw6.3_RG_*. No significant differences in leaf morphological characteristics, the number of flowers per inflorescence, the number of fruits per inflorescence, flowering time, and node below the first flower cluster were observed between the two NILs (Tables S12–S15).

A flower and leaf removal experiment was conducted to determine whether *fw6.3* had direct effects on fruit weight or whether the effect of *fw6.3* on fruit weight was mediated via a sink–source dependent mechanism. Each plant of the two NILs was pruned to four inflorescences, with three fruits per inflorescence and two leaves between each pair of inflorescences. The fruit weight, fruit diameter, and fruit length were higher in mature fruit containing the *fw6.3_HG_* allele than in mature fruit containing the *fw6.3_RG_* allele; however, no significant difference was observed in the fruit shape index between fruits with the *fw6.3_HG_* allele and with the *fw6.3_RG_* allele (Table 2). Similar findings were obtained in control plants in which no leaves and flowers were removed (Table 2), which indicates that the effect of *fw6.3* on fruit weight was direct.

### 2.7. SSC of Fruit

A locus affecting SSC has been reported to overlap the *fw6.3* locus in previous studies [18,21,22]. However, whether these represent two single-trait loci in close linkage or a single pleiotropic locus remains unclear. The *fw6.3* locus was fine-mapped to a 62.6 kb interval that was lacking in sequence variation, with the exception of an SNP in the *SP* gene between RG and HG. SSC data collected from 2020 to 2022 revealed that the SSC of *fw6.3_HG_* and *fw6.3_LA1589_* fruits was significantly higher than that of *fw6.3_RG_* fruits (Table 3 and Table S8), which suggests that the *fw6.3* locus also affected the SSC of fruit. The SSC of the fruit of *fw6.3_HG_* plants was also higher than that of *fw6.3_RG_* plants in the flower and leaf removal experiment (Table 3).

## 3. Discussion

Tomato fruit weight is a key yield component. Clarifying the genetic basis of fruit weight is important for tomato breeding programs and studies of the molecular regulation of fruit development. Tomato fruit weight is a quantitative trait. Approximately 30 loci affecting tomato fruit weight have been identified to date [1,2,18,23,24]. However, only six of these loci have been fine-mapped and cloned [4,5,6,10,11]. In this study, *fw6.3* was identified and located on the long arm of chromosome 6. This locus explained 11.8% of the variation in fruit weight (Figure 1, Table S1). This locus was fine-mapped to a 62.6 kb interval that contains seven genes (Figure 2, Table S8). According to the analysis of the sequence polymorphism, only the *SP* gene contained an SNP that results in an amino acid substitution, and this same SNP has been identified in previous studies [20,25]. Thus, *SP* was the putative candidate gene for the *fw6.3* locus. Transgenic complementation assays will be performed to confirm this possibility.

Heterosis has been widely used in breeding programs to enhance yield. Dominance and overdominance effects are major contributors to heterosis. Genes showing overdominance are of interest to both breeders and scientists. However, only a few genes have been demonstrated to show single-gene overdominance [26]. A rapeseed mutant (*sca*) with a point mutation in *Aux/IAA7* (*BnaA3.IAA7*) shows semi-dwarfism and has a compact architecture. The heterozygote +/*sca* exhibits strong yield heterosis, which is driven by the integration of overdominance in 1000-seed weight and dominance in seeds per silique and siliques per plant [27]. The tomato mutants *compound inflorescence* (*s*) and *s2* are characterized by extensive inflorescence branching and low fruit set [28,29]. The *S* gene encodes a WUSCHEL-homeobox (WOX) protein [29]. Two loci, *j2^TE^* (*jointless-2*) and *ej2^w^* (*enhancer-of-jointless2*) affect the phenotype of *s2*. *J2* and *EJ2* both encode MADS-box proteins [28]. Heterozygotes (*s*/+) show yield heterosis, including a greater number of flowers per plant and fruits per plant, which stems from the reduced number of inflorescence branches and normal fruit set. Hybrid lines *j2^TE^ ej2^w^*/+ also show yield heterosis [28]. The tomato *SINGLE FLOWER TRUSS (SFT)* gene shows single-gene overdominance. In the *sp* mutant background, *sft* homozygous mutants exhibit delayed flowering and have few flowers, and *sft*/+ heterozygotes show strong yield heterosis due to their greater number of inflorescences and fruits. This heterosis is eliminated when plants harbor a normal *SP* gene [30]. Both *SFT* and *SP* are members of the *CENTRORADIALIS/TERMINAL FLOWER 1/SELF-PRUNING* gene family. SFT is the tomato flowering hormone florigen, and SP is an antagonist of SFT [31]. Dosage effects of the heterozygous mutations in the florigen pathway components are thought to fine-tune the balance between the florigen and its antagonist, which enhances tomato yield [32,33]. In this study, the large-fruit allele of *fw6.3* was overdominant to the small-fruit allele, suggesting that fw6.3 could be useful for yielding heterosis breeding (Table 1, Tables S1 and S10). *SP* was the putative candidate gene of *fw6.3*. In previous studies, *sft* homozygous mutants have been shown to have larger fruits than wild-type plants, and *sft*/+ heterozygotes show an additive effect for fruit weight in a *sp* mutant background [30]. Additional studies are needed to clarify the roles of *SP* and *SFT* in controlling fruit weight at the molecular level and the molecular mechanism underlying fruit weight heterosis.

Higher yield and quality are the main goals of tomato breeding programs. Several studies have reported a negative relationship between fruit weight and SSC, which are key components of yield and quality, respectively [34,35]. The identification of QTLs that mediate increases in both fruit weight and SSC is important for promoting increases in the yield and quality of tomato via marker-assisted selection. *fw6.3* and *ssc6.1* are co-localized to the end of chromosome 6 and mediate increases in fruit weight and SSC, respectively [23,36]. Whether the co-localization of *fw6.3* and *ssc6.1* and their effects on fruit weight and SSC stem from pleiotropic effects of the same gene or the tight linkage of different QTLs remains unclear. In our study, both *fw6.3* and *ssc6.1* were fine-mapped to a 62.6 kb interval, and no sequence variation was detected in this 62.6 kb interval, with the exception of an SNP in the *SP* gene between RG and HG (Figure 2b, Table S9). Thus, *SP* was assumed to be the putative candidate gene of *fw6.3* and *ssc6.1*. The weak *SP* mutant *sp*-5732 has a higher Brix value, which indicates a higher sugar content, and Brix yield than the strong *SP* mutant *sp-classic* [25]. According to our findings, *sp-classic* is the putative small-fruit allele of *fw6.3*. Therefore, the *SP* gene might positively regulate fruit weight and SSC, which suggests that it could be a useful target gene for tomato breeding programs via marker-assistant selection or gene editing.

## 4. Conclusions

We identified a major-effect locus, *fw6.3*, that affects tomato fruit weight, and the large-fruit allele of *fw6.3* was overdominant to the small-fruit allele in fruit weight. The SSC of tomato fruit is also increased by the *fw6.3* locus. Fine mapping of the *fw6.3* locus revealed that it was on chromosome 6 in a 62.6 kb interval containing seven genes. The candidate gene most strongly associated with tomato fruit weight was *Solyc06g074350*, which is the *SP* gene. Our findings will aid the cloning of the *FW6.3* gene as well as the molecular breeding of tomato plants with higher yield and quality.

## 5. Materials and Methods

### 5.1. Plant Materials

Seeds of Rio Grande (*S. lycopersicum*), LA1589 (wild species; *S. pimpinellifolium*), and HGL (a line derived from a cross between HG and LA1589) were obtained from the laboratory of Esther van der Knaap at The Ohio State University [15]. HGL is an offspring of plant 11S74-3 that was used in the fine-mapping of *fw11.3* and derived from a cross between Howard German (HG) and LA1589 [6]. HGL contains the large fruit allele of *FW11.3*, which was detected with the functional marker HP32 of *FW11.3*. Both HGL and LA1589 show indeterminate growth, whereas Rio Grande is characterized by determinate growth. A total of 388 plants in an F_2_ population derived from a cross between HGL and RG were grown in a plastic greenhouse in Shunyi District, Beijing, China, in the spring of 2014. The recombinants, heterozygous plants, and progeny test populations for fine-mapping of the *fw6.3* locus were grown in a plastic greenhouse in the spring in Shunyi District, Beijing, China, from 2015 to 2022. RG was used as the recurrent parent to develop NIL-*fw6.3_HG_*, NIL-*fw6.3_LA1589_*, and NIL-*fw6.3_RG_*. These NILs were grown in a plastic greenhouse in the spring in Shunyi District and Haidian District, Beijing, China, from 2019 to 2022. The pedigrees of the materials used in this study are shown in Tables S16–S18.

### 5.2. Phenotypic Analysis

Measurements of the fruit weight were taken on a total of 10 red, ripe tomato fruits from each plant. A digital pocket refractometer PAL-1 (Atago Co. Ltd., Tokyo, Japan) was used to measure the SSC of the 10 tomato fruits, and SSC values were expressed in Brix. Digital vernier calipers DL3944 (Deli Group Co., Ltd, Ningbo, China) were used to measure the length and diameter of tomato fruits at various stages of fruit development. When tomato fruits in the first cluster were mature, several morphological characteristics of the eighth leaf of each plant were measured. In the flower and leaf removal experiments, each plant in the NIL-*fw6.3_HG_* and NIL-*fw6.3_RG_* groups was pruned to four inflorescences, with three fruits per inflorescence and two leaves between each pair of inflorescences. Several characteristics of the tomato fruits were measured at the red ripening stage.

### 5.3. QTL-Seq Analysis of Fruit Weight

QTL-seq of an F_2_ population derived from a cross between RG and HGL was used to identify loci that affect tomato fruit weight. F_2_ plants were organized from small to large, based on the weight of tomato fruits. The SFP comprised the DNA of 20 plants with the smallest fruits, and the LFP comprised the DNA of 20 plants with the largest fruits. The cetyltrimethylammonium bromide method was used to extract genomic DNA from F_2_ plants [37]. Genomes of RG, HGL, the SFP, and the LFP were resequenced (10× genome coverage) using the Illumina HiSeq 2000 PE100 platform (Illumina, San Diego, CA, USA). After removing low-quality reads, Burrows–Wheeler Aligner software was used to align the clean reads to the tomato reference genome (version SL4.0) [38]. SNPs throughout the tomato reference genome were called using SAMtools and BCFtools [39,40]. 

SNP-indexes of the SFP and LFP were calculated using the SNPs of RG as a reference. The average SNP-index across the genome was calculated via sliding window analysis, with a window size of 1 Mb and step size of 10 kb [1]. The difference between the SNP-index of the SFP and the SNP-index of the LFP was used to calculate Δ(SNP-index). QTLs with |Δ(SNP-index)| values greater than 0.3 were assumed to be candidate QTLs affecting tomato fruit weight [41].

### 5.4. Development of Molecular Markers and Genotyping

Insertions, deletions, and SNPs among RG, HGL, and LA1589 identified using the resequencing data obtained from our study or from the SGN (https://solgenomics.net/, accessed on 22 July 2017) were used to develop polymerase chain reaction-based markers. Details of the markers used in this study are shown in Table S19. Markers in the regions of the candidate QTLs identified by QTL-seq analysis were used to genotype all F_2_ plants. The significance of the association of the markers with variation in tomato fruit weight in the F_2_ population was assessed via a one-way analysis of variance. In the analysis of gene action, the degree of dominance was calculated using the d/a ratio, where d = Aa − (AA + aa)/2 and a = (AA − aa)/2 [15]; AA is the mean value for the homozygous HGL allele, aa is the mean value for the homozygous RG allele, and Aa is the mean value for the heterozygous genotypes. Following the classification criteria employed by Burke et al. [42] and Doms et al. [43], the mode of gene action of the HGL allele at each locus was categorized as follows: underdominant ≤ −1.25 < recessive ≤ −0.75 < partially recessive ≤ −0.25 < additive < 0.25 ≤ partially dominant < 0.75 ≤ dominant < 1.25 ≤ overdominant. Multiple regression analysis was used to evaluate the amount of phenotypic variation explained per QTL, and the most significant markers per QTL (according to R^2^ values) were used as explanatory variables [44].

### 5.5. Fine Mapping of the fw6.3 Locus

The *fw6.3* locus was fine-mapped using recombinants with crossover sites around the *fw6.3* locus. In the progeny tests of each recombinant, two markers in the heterozygous region around the *fw6.3* locus were used to genotype offspring seedlings. Phenotypic analysis was conducted on a set of homozygous plants carrying the RG allele, as well as the HGL allele or the LA1589 allele, grown in a greenhouse. Several recombinants and heterozygous plants (if no recombinants were found) were grown in a greenhouse and self-crossed to conduct progeny tests for the next generation of progeny. Searches of the available tomato genome annotation database (ITAG release 4.0) in the SGN (https://solgenomics.net/) were conducted to identify putative genes in the region near the *fw6.3* locus.

## Figures and Tables

**Table 1:** Results of the gene action analysis of *fw6.3*.

PopulationID	HP865 ^a^	JP67	JP73	HMT	HP1325	HP1337	Plant N ^b^	Ave FW ^c^	d/a ^d^	*p*-Value
18N301	1	1	1	1	1	1	12	91.55 ± 15.19	1.73	8.90 × 10^−5 e^
(BC2S4)	1	1	2	2	1	1	12	121.29 ± 15.26		0.0026 ^f^
	1	1	3	3	1	1	12	113.31 ± 16.22		0.23 ^g^
21N1175	1	1	1	1	1	1	12	70.20 ± 12.59	1.38	4.64 × 10^−10 e^
(BC4S6)	1	1	1	2	1	1	12	130.25 ± 15.21		5.17 × 10^−9 f^
	1	1	1	3	1	1	12	120.62 ± 14.15		0.12 ^g^
21N1177	1	1	1	1	1	1	12	91.89 ± 10.02	1.27	4.01 × 10^−9 e^
(BC7S4)	1	1	2	2	1	1	12	134.22 ± 12.05		3.35 × 10^−9 f^
	1	1	3	3	1	1	12	129.12 ± 9.27		0.26 ^g^

^a^ Marker score: 1 homozygous for *S. lycopersicum* c.v. Rio Grande alleles (RG); 2 heterozygous; 3 homozygous for *S. lycopersicum* c.v. Howard German alleles (HG); ^b^ number of tomato plants; ^c^ AFW: average fruit weight (g), data were given as mean ± standard deviation (SD). ^d^ d/a = degree of dominance. The Student’s *t*-test was used to compare the means between group 1 and group 2 (^e^), between group 1 and group 3 (^f^), between group 2 and group 3 (^g^).

**Table 2:** Phenotypic data of the *fw6.3* NILs in the flower and leaf removal experiment.

Population ID	Genotype	Plant N	Ave FW ^a^	*p*-Value ^b^	Ave Diam ^c^	*p*-Value	Ave Length ^d^	*p*-Value	Fruit Shape Index	*p*-Value
21N1175	*fw6.3_RG_*	12	70.20 ± 12.59	5.17 × 10^−9^	46.25 ± 6.97	4.83 × 10^−23^	63.72 ± 9.41	3.03 × 10^−20^	1.39 ± 0.14	0.09
(Control)	*fw6.3_HG_*	12	120.62 ± 14.15		54.64 ± 3.97		74.22± 5.46		1.36 ± 0.10	
21N1175	*fw6.3_RG_*	10	92.30 ± 13.51	3.38 × 10^−7^	52.06 ± 5.08	7.53 × 10^−15^	70.17 ± 6.14	3.39 × 10^−20^	1.35 ± 0.09	0.04
(Flower removal)	*fw6.3_HG_*	12	138.09 ± 14.98		59.09 ± 5.06		81.32± 4.97		1.38 ± 0.10	

^a^ Ave FW: average ripe fruit weight (g), data were given as mean ± SD. ^b^ *p*-Value: Student’s *t*-test. ^c^ Ave Diam: average ripe fruit diameter (mm). ^d^ Ave Length: average ripe fruit length (mm).

**Table 3:** Fruit SSC of the *fw6.3* NILs.

Population ID	Genotype	Plant N	Ave SSC ^a^	*p*-Value ^b^
20N198	*fw6.3_RG_*	24	3.85 ± 0.38	2.22 × 10^−8^
	*fw6.3_HG_*	21	4.59 ± 0.35	
20N199	*fw6.3_RG_*	28	3.92 ± 0.37	5.21 × 10^−8^
	*fw6.3_HG_*	28	4.69 ± 0.52	
21N1175(Control)	*fw6.3_RG_*	12	4.19 ± 0.38	1.37 × 10^−5^
	*fw6.3_HG_*	12	4.96 ± 0.11	
21N1175 (Flower removal)	*fw6.3_RG_*	10	3.94 ± 0.34	2.14 × 10^−5^
	*fw6.3_HG_*	12	4.60 ± 0.21	

^a^ Ave SSC: average soluble solids content, data were given as mean ± SD. ^b^ *p*-Value: Student’s *t*-test.

## Data Availability

Not applicable.

## Supplementary Materials

The following supporting information can be downloaded at: https://www.mdpi.com/article/10.3390/plants12112065/s1, Figure S1. Changes in fruit size during fruit development in the year 2020 Table S1. Markers associated with fruit-weight QTLs; Table S2. Progeny testing and fine mapping of the *fw6.3* locus in the spring of 2015; Table S3. Progeny testing and fine mapping of the *fw6.3* locus in the spring of 2016; Table S4. Progeny testing and fine mapping of the *fw6.3* locus in the spring of 2017; Table S5. Predicted genes in the *fw6.3* candidate region (248.8 kb interval); Table S6. Progeny testing of the population from HG and LA1589; Table S7. Progeny testing and fine mapping of the *fw6.3* locus in the spring of 2021; Table S8. Progeny testing and fine mapping of the *fw6.3* locus in the spring of 2022; Table S9. Predicted genes in the *fw6.3* candidate region (62.6 kb interval); Table S10. Results of the gene action analysis of the *fw6.3* locus; Table S11. The size and mass of ripe fruit in two *fw6.3* near-isogenic lines at various stages of fruit development; Table S12. Leaf morphological characteristics of the *fw6.3* NILs; Table S13. Ripe fruit of the *fw6.3* NILs; Table S14. Summary of the inflorescences and fruits of the *fw6.3* NILs; Table S15. Flowering time and flowering node of the *fw6.3* NILs; Table S16. Pedigrees of materials derived from the cross between RG and HGL; Table S17. Pedigrees of *fw6.3* NILs; Table S18. Pedigrees of the materials derived from the cross between RG and LA1589; Table S19. DNA markers used in this study.
